# Food‐Borne Diseases in Bangladesh: First Assessment of Knowledge, Attitudes, and Practices of Farmers

**DOI:** 10.1155/vmi/3617712

**Published:** 2026-06-30

**Authors:** Md Jisan Ahmed, Prajwal Bhandari, Md Ismile Hossain Bhuiyan, Ritu Chalise, Soumik Sarker, Md Sazzad Bin Yousuf, Md Easin, Md Rayhan Ullah Rahi, Hridoy Chakrabarty, Ajoy Paul, Prottoy Basak, Rahat Nahiyan Ramim, Delower Hossain

**Affiliations:** ^1^ Association of Coding, Technology, and Genomics (ACTG), Dhaka, 1207, Bangladesh; ^2^ Department of Pathology, Faculty of Animal Science and Veterinary Medicine, Sher-e-Bangla Agricultural University (SAU), Dhaka, 1207, Bangladesh, sau.edu.bd; ^3^ Department of Microbiology and Parasitology, Faculty of Animal Science and Veterinary Medicine, Sher-e-Bangla Agricultural University (SAU), Dhaka, 1207, Bangladesh, sau.edu.bd; ^4^ Department of Animal Production and Management, Faculty of Animal Science and Veterinary Medicine, Sher-e-Bangla Agricultural University (SAU), Dhaka, 1207, Bangladesh, sau.edu.bd; ^5^ Department of Medicine and Public Health, Faculty of Animal Science and Veterinary Medicine, Sher-e-Bangla Agricultural University (SAU), Dhaka, 1207, Bangladesh, sau.edu.bd

**Keywords:** Bangladesh, farmers, food-borne disease, KAP, One Health, zoonoses

## Abstract

**Background:**

Food‐borne diseases are significant threats to global public health, particularly in developing countries such as Bangladesh, where zoonotic food‐borne pathogens cause substantial illnesses, death, and economic losses.

**Objective:**

This study aims to assess the knowledge, attitudes, and practices (KAPs) of livestock farmers in Bangladesh toward food‐borne zoonotic diseases and identify key factors influencing their awareness and behavior.

**Methods:**

A cross‐sectional survey was conducted from April to September 2025 among 523 farmers from eight divisions of Bangladesh using a structured questionnaire. The participants were over 18 years old, owned at least two livestock or 15 poultry birds, and reported animal diseases to veterinary hospitals.

**Results:**

Among them, 74.4% were male, most were aged 32–45 years, and 50.7% had an education above secondary level. Three‐quarters of the participants (74.4%) were aware of tuberculosis, followed by salmonellosis (68.6%), *E. coli* infection (65.0%), anthrax (65.0%), and clostridial infections (41.5%). A total of 88.9% of respondents identified ingestion of raw or undercooked meat as the primary cause, followed by contaminated water (79.4%) and the consumption of raw or unpasteurized milk and milk products (75.7%). However, only 8.8% of the farmers demonstrated good knowledge, defined as adequate understanding of food‐borne diseases, including their transmission, symptoms, and prevention, and 38.2% exhibited good management practices, with male farmers showing comparatively higher KAP scores. Univariate regression showed that variables such as sex, education, age, marital status, and training significantly influenced KAP. Despite a generally positive attitude, reflecting a willingness to prevent food‐borne diseases and recognition of their health importance, farmers lacked adequate awareness of and adherence to safe practices to prevent zoonotic diseases.

**Conclusion:**

This study underscores the need for enhanced training, community awareness, and integrated One Health collaboration to improve food safety and reduce zoonotic risk in Bangladesh.

## 1. Introduction

Food‐borne diseases (FBDs) are a major global public health concern, causing significant illness, death, and economic losses [1]. A significant subset consists of zoonotic diseases, hereafter referred to as food‐borne zoonoses [2]. FBDs are transmitted from animals to humans through contaminated food, direct or indirect contact, or environmental exposure [3]. They are caused by bacteria, viruses, parasites, and prions, which contaminate food and cause illness when ingested; bacterial and viral pathogens may infect the host or produce toxins, parasitic organisms establish infection after consumption of contaminated food, and prions are infectious proteins associated with fatal neurodegenerative diseases [4, 5]. The consumption of contaminated animal products is a leading cause of human infections and poses a significant risk to both individual and community health [6].

Zoonotic diseases, such as tuberculosis, salmonellosis, avian influenza, brucellosis, and leptospirosis, pose serious threats to both human and animal health, causing morbidity, mortality, and economic losses through reduced production of meat, milk, and wool, while also compromising food safety [7]. According to the WHO, more than 200 diseases, ranging from mild gastrointestinal infections to severe conditions such as cancer, are linked to the consumption of unsafe food contaminated with harmful microorganisms or chemicals [8]. Globally, an estimated 600 million people fall ill, and 420,000 die each year due to FBDs, accounting for a loss of 33 million disability‐adjusted life years [9, 10]. In some industrialized countries, the percentage of the population suffering from FBDs each year is estimated to be up to 30% [11].

FBDs often arise from improper food handling, whether at home or in commercial environments, and contamination can occur at any stage of the food chain, including production, processing, distribution, and consumption [12, 13]. The contributing factors include poor sanitation, weak regulatory and enforcement systems across the food supply chain (from production to consumption), inadequate food safety laws, insufficient equipment, limited financial resources, and insufficient education, particularly in relation to safe food handling practices, hygiene, and awareness of FBD risks [14, 15]. Therefore, FBDs remain a significant global public health concern, particularly in developing countries [11].

The growing global demand for animal‐source foods such as meat, milk, eggs, and fish, driven by rapid population growth, urbanization, increasing incomes, and dietary shifts toward high‐protein diets, has intensified animal production and global food processing, increasing the risk of contamination along the farm‐to‐fork chain [6, 16]. Animal foods are particularly associated with FBDs because of their high pathogen load, natural toxins, adulterants, and other contaminants [17, 18]. Food‐producing animals such as cattle, poultry, pigs, and fish act as major reservoirs for zoonotic pathogens, linking increased consumption of animal‐derived foods to a greater risk of FBDs in humans [19–21].

In developing countries, including Bangladesh, the public health impact of zoonotic food‐borne pathogens is increasingly recognized. However, FBDs are often underreported due to complex reporting procedures, with only those seeking medical attention being recorded by public health authorities [20]. Although surveillance is limited, it is estimated that approximately 30 million people (approximately 17% of the total population) in Bangladesh are affected by FBDs annually [12]. Improving food safety requires understanding the knowledge, attitudes, and practices (KAPs) of target populations, which helps design educational interventions to promote safe dietary habits, reduce disease risk, and positively influence community health [22, 23]. To date, no formal study has evaluated KAPs regarding food safety among farmers in Bangladesh, highlighting the need for such research. Farmers play a critical role in the food production chain, as they are directly involved in the rearing, handling, and initial processing of animal‐source foods. Their KAPs can significantly influence the risk of FBDs at the source, thereby impacting both public health and food safety. Therefore, focusing on farmers provides essential insights into upstream risk factors and potential intervention points. Accordingly, the primary goal of this research was to explore farmers’ KAPs regarding FBDs and identify factors influencing their KAPs to inform targeted educational and preventive interventions.

## 2. Materials and Methods

### 2.1. Study Area

The study was conducted across eight divisions, including Dhaka, Chattogram, Rajshahi, Khulna, Barishal, Sylhet, Rangpur, and Mymensingh, in Bangladesh from April 1 to September 30, 2025. The country is largely composed of low‐lying alluvial floodplains formed by the Ganges–Brahmaputra–Meghna River system, with limited hilly regions mainly in the southeast (Chattogram) and northeast (Sylhet). Central divisions such as Dhaka and Mymensingh consist of dense riverine plains, while western regions like Rajshahi and Rangpur include relatively elevated and drier tracts. Coastal divisions, including Khulna and Barishal, are characterized by deltaic plains, mangrove forests, and vulnerability to cyclones and salinity intrusion, whereas eastern divisions experience higher rainfall and humid conditions [24]. Bangladesh has a tropical monsoon climate marked by high temperatures, heavy rainfall, and high humidity, with three dominant seasons: a hot summer, a rainy monsoon, and a cool, dry winter [25]. Regional climatic variation is relatively limited due to the country’s flat terrain, although the eastern regions receive significantly more precipitation than the relatively drier northwest.

### 2.2. Study Population and Selection of Farmers

The study population comprised livestock and poultry farmers from the selected division of Bangladesh who owned a minimum of two animals or 15 birds at the time of data collection. Farmers from various districts within the selected divisions of Bangladesh were randomly selected based on age and livestock or poultry ownership. The selection of participants was not proportional to the distribution of herds across geographical regions. The eligibility criteria for participant selection were defined as follows: (i) farmers aged ≥ 18 years residing within the study area, irrespective of sex; (ii) willingness to participate in the survey, with verbal informed consent obtained before the interview; (iii) inclusion of only one respondent from each household; (iv) livestock farmers owning at least two animals, including cattle, sheep, goats, buffalo, or pigs, or poultry farmers rearing a minimum of 15 birds, such as chickens, ducks, pigeons, or quail; and (v) farmers who had previously reported animal or poultry diseases to a veterinary hospital, ensuring prior engagement with animal health services and practical experience in disease management. Farmers were excluded from participation if they were younger than 18 years, declined to participate in the study, or were unable to provide reliable responses during the interview.

### 2.3. Sample Size

As the prevalence of KAPs related to FBDs in the target population was unknown, an expected prevalence of 50% was assumed to ensure maximum sample size estimation. Accordingly, the minimum required sample size was calculated using the standard single population proportion formula described by the Thrusfield formula [26]:

(1) n=Z2pqd2.

In the formula, *n* represents the minimum required sample size, *Z* denotes the standard normal deviate corresponding to a 95% confidence level (1.96), *p* refers to the assumed proportion of farmers possessing specific KAP attributes (50%), *q* represents 1 − *p*, and *d* indicates the desired precision level or margin of error (0.05). Based on these assumptions, the calculated sample size was

(2) n=1.962X0.5X10.5−0.052=384.16.

Thus, the minimum required sample size was determined to be 384 participants. Nevertheless, to enhance the statistical precision and representativeness of the study, the final sample size was increased to 523 respondents, distributed across the administrative divisions of Bangladesh as follows: Dhaka (*n* = 53), Chattogram (*n* = 82), Rajshahi (*n* = 47), Khulna (*n* = 61), Barishal (*n* = 65), Sylhet (*n* = 77), Rangpur (*n* = 70), and Mymensingh (*n* = 68). The inclusion of a sample size larger than the minimum required improved the robustness and reliability of the study’s findings while maintaining practical feasibility.

### 2.4. Questionnaire Design and Administration

A well‐structured questionnaire comprising 40 items was designed to collect data from farmers, focusing on their perceptions and preventive measures regarding FBDs (Supporting File 1). The questionnaire was developed primarily through an extensive review of previously published studies on KAPs regarding FBDs [27–30]. A pilot test was conducted with 20 students to evaluate the clarity and suitability of the questionnaire items and to estimate the time needed for completion. The questionnaire was modified based on the pilot study findings and further adjusted to align the questions with Bangladesh’s cultural context. The finalized questionnaire primarily comprised closed‐ended questions and was structured into five distinct sections. The first section focused on collecting sociodemographic characteristics of the participants, including age, sex, and educational status. The second section assessed farmers’ knowledge of FBDs, with questions on common FBDs, modes of transmission, and preventive strategies. The third section explored farmers’ attitudes, focusing on their perceptions of zoonoses, food safety, and preventive practices. The fourth section examined the preventive measures farmers adopt to protect against FBDs. Finally, the fifth section addressed farmers’ access to information and resources on FBDs. The questionnaire was originally prepared in English and subsequently translated into Bengali, the local language, to facilitate participant comprehension. Data were collected through face‐to‐face interviews to ensure accuracy and completeness of responses. The translated version was reviewed and validated by the study team to confirm its accuracy, clarity, and contextual appropriateness.

### 2.5. Data Collection Tools

Efficient and systematic data collection was conducted via an online survey in the Kobo Toolbox (version 2.023.37, Open Data Kit software, KoboCollect, https://kf.kobotoolbox.org). All data collectors were trained in using Kobo Toolbox to ensure consistency, standardization, and accuracy during the data collection process.

### 2.6. Data Collection

To standardize data collection, 10 veterinary students (undergraduate) from Sher‐e‐Bangla Agricultural University were trained over 2 days in the study protocol, interview procedures, and questionnaire administration. Farmers were approached through local contacts, farm visits, and community‐based networks, and their participation was requested voluntarily. Interviews were conducted in person at locations convenient for the farmers, such as their homes, farms, or nearby community settings, ensuring minimal disruption to their daily farming activities. Data were collected over 6 months through face‐to‐face interviews with farmers using a structured and pretested questionnaire that did not interfere with their farming activities. Participants were informed that all responses would be treated confidentially and anonymously, thereby promoting honest reporting and reducing the likelihood of response bias. On average, each interview required approximately 20–25 min to complete.

A structured questionnaire was administered to assess respondents’ knowledge of FBDs. Participants were initially asked whether they were familiar with the term “food‐borne diseases.” For respondents who answered “no,” a brief, standardized definition describing FBDs as diseases transmitted through the consumption of contaminated animal‐derived food products was provided before continuing to subsequent questions. The diseases included in the questionnaire were selected based on their public health importance, relevance to the regional context, and established association with food‐borne transmission as reported in the literature. The “none of the above” option was not included because the questionnaire was designed to evaluate awareness of a predefined set of the most common and significant FBDs in the study area.

### 2.7. Scoring

A systematic approach was used to determine individuals’ KAPs following previously published literature [27, 31]. Each correct or positive response was scored as 1, whereas incorrect or negative responses were scored as 0. The detailed classification of correct and incorrect answers is provided in Supporting File 1 (Knowledge). An overall KAP score was computed for each participant by aggregating positive responses across all relevant items and converting the total into a percentage. A score of 100% reflected complete adherence to the recommended KAP attributes. The median score (50%) was used as a cutoff to categorize this continuous variable and as the dependent variable in simple and multiple regression analyses [32]. Farmers scoring above the median were considered to have good knowledge, positive attitude, and good practices. Knowledge was assessed on the basis of respondents’ ability to correctly identify FBDs, their transmission routes, symptoms, and preventive measures, using a series of structured questions. Attitude was evaluated using a set of Likert‐scale statements reflecting respondents’ perceptions and beliefs regarding the seriousness, preventability, and public health importance of FBDs. A positive attitude was characterized by responses demonstrating awareness of disease risks, agreement with preventive measures, and willingness to adopt safe practices, whereas a negative attitude indicated low perceived risk, disagreement with preventive measures, or a lack of concern about food safety. Moreover, practice‐related questions assessed whether respondents followed recommended preventive measures against FBDs. Depending on the score, knowledge was categorized as poor, moderate, or good and attitude as negative, uncertain, or positive. Scores of 0%–49% were classified as poor/negative, 50%–79% as moderate/uncertain, and 80%–100% as good/positive [32]. However, practice‐related questions were included to evaluate whether preventive measures against FBDs were applied appropriately or inappropriately. For practice, scores above 80% were classified as acceptable, appropriate, or good, whereas scores below 80% were considered unacceptable, inappropriate, or poor.

### 2.8. Data Analysis

The collected responses were exported from Kobo Toolbox to Microsoft Excel and checked for completeness through data filtering. Responses with complete data across all the questions were considered valid and subsequently imported into R software (v4.5.0) for analysis. R packages such as “*gtsummary*” were used to summarize sociodemographic data and produce reproducible descriptive tables [33]. Categorical variables, such as sex and education level, were presented as frequencies and percentages. Similarly, the “*ggplot*” package was used to generate graphs [34]. Associations between KAP levels and sociodemographic characteristics were initially examined using Pearson’s chi‐square (*χ* ^2^) test implemented in the *tidyverse* package. To identify factors associated with KAP outcomes, univariable ordinal logistic regression analyses were first performed to estimate crude associations. Variables showing potential associations were subsequently included in multivariable ordinal logistic regression models, fitted using the ordinal package (*clm*) [35]. Initially, univariate ordinal regression models were fitted for each predictor variable separately, using a cumulative link function (logit). Subsequently, a multivariate ordinal regression model was constructed by including all predictor variables simultaneously to obtain adjusted estimates. The general model structure was as follows:

Knowledge level ∼ gender + age + education + marital status + family size + training + religion. Similar model structures were applied for attitude and practice outcomes. All outcome variables (knowledge, attitude, and practice levels) were treated as ordered factors, with categories arranged from lowest to highest (e.g., poor ⟶ moderate ⟶ good for knowledge; negative ⟶ uncertain ⟶ positive for attitude; poor ⟶ good for practice). All variables considered theoretically relevant or identified a priori were included in the multivariate regression model, regardless of their statistical significance in the univariate analysis, to control for potential confounding effects. A *p* value of less than or equal to 0.05 (*p* ≤ 0.05) was considered statistically significant for all analyses. Moreover, multicollinearity among predictor variables was assessed using the variance inflation factor (VIF), with generalized VIF (GVIF) values adjusted for degrees of freedom, where applicable. Model adequacy was evaluated using the Hosmer–Lemeshow goodness‐of‐fit test. A *p*‐value greater than 0.05 was considered indicative of acceptable model fit.

### 2.9. Ethical Approval

The study adhered to established ethical guidelines for research involving human participants, ensuring the protection of their rights, welfare, and personal dignity. Ethical approval for the study was obtained from the Microbiology and Parasitology of Sher‐e‐Bangla Agricultural University Ethical Review Board (Reference No. SAU‐MIPA‐ERB/2025/020). Prior to data collection, informed consent was obtained from all participants after clearly explaining the objectives and procedures of the study. Participants were informed of their voluntary participation and their right to refuse participation or withdraw from the study at any stage without any consequences. Data collection commenced only after consent had been provided by the participants.

## 3. Results

### 3.1. Demographic Characteristics of the Participants

A total of 523 farmers were interviewed in this study. The majority of the participants were male (*n* = 389, 74.4%), and more than half (*n* = 286, 54.7%) were in the 18–30 age group. With respect to educational status, 265 participants (50.7%) had studied beyond the secondary level. A significant majority, 414 (79.2%) farmers, had medium‐sized families with 4–6 members, and 280 (53.5%) were married. Only 119 (22.8%) farmers had received training on food safety and hygiene. Regarding religion, the majority of participants (*n* = 422, 80.7%) were Muslim (Table 1).

**TABLE 1:** Sociodemographic data of respondents (*n* = 523), including frequency, percentage distribution, and 95% confidence intervals (CI), among adult respondents aged ≥ 18 years.

Variables		*N* = 523^1^	95% CI
Age	18–30 years	286 (54.68%)	50%, 59%
	31–45 years	144 (27.53%)	24%, 32%
	> 45 years	93 (17.78%)	15%, 21%
Gender	Female	134 (25.62%)	22%, 30%
	Male	389 (74.38%)	70%, 78%
Education	Illiterate	28 (5.35%)	3.7%, 7.7%
	Primary	75 (14.34%)	12%, 18%
	Secondary	155 (29.64%)	26%, 34%
	Higher	265 (50.67%)	46%, 55%
Family members	1–3 persons	59 (11.28%)	8.8%, 14%
	4–6 members	414 (79.16%)	75%, 83%
	> 6 persons	50 (9.56%)	7.2%, 12%
Marital status	Married	280 (53.54%)	49%, 58%
	Others	6 (1.15%)	0.47%, 2.6%
	Single	237 (45.32%)	41%, 50%
Training on food safety and hygiene	No	404 (77.25%)	73%, 81%
	Yes	119 (22.75%)	19%, 27%
Religion	Hindu	96 (18.36%)	15%, 22%
	Muslim	422 (80.69%)	77%, 84%
	Others	5 (0.96%)	0.35%, 2.3%

Abbreviation: CI = confidence interval.

^1^ *n* (%).

### 3.2. Knowledge of Farmers Toward Food‐Borne Zoonoses

Among the 523 participants, 472 (90.3%) had heard about FBDs (Table 2). Among the participants, awareness of tuberculosis was the highest (*n* = 389, 74.4%), followed by salmonella infection (*n* = 359, 68.6%), *E. coli* infection (*n* = 340, 65.0%), anthrax (*n* = 340, 65.0%), and clostridial infections (*n* = 217, 41.5%). Regarding disease transmission, most participants identified the consumption of raw or undercooked meat as the primary route (*n* = 465, 88.9%), followed by contaminated water (*n* = 415, 79.4%) and raw or unpasteurized milk and milk products (*n* = 396, 75.7%). Similarly, 445 farmers (85.1%) acknowledged that reporting unusual animal or human disease outbreaks helps early detection and prevention. A large proportion of participants (*n* = 458, 87.6%) believed that vaccinating animals helps control FBDs at both the farm and community levels.

**TABLE 2:** Farmer’s response on knowledge of food‐borne diseases in Bangladesh (*n* = 523), presented as frequencies and percentages.

Variables	Yes	No
1. Have you heard of “food‐borne zoonotic diseases?”	472 (90.25%)	51 (9.75%)
2. Name of food‐borne zoonoses?		
Novo virus	29 (5.54%)	494 (94.46%)
Salmonellosis	359 (68.64%)	164 (31.36%)
Campylobacteriosis	139 (26.58%)	384 (73.42%)
Clostridial infection	217 (41.49%)	306 (58.51%)
Listeriosis	85 (16.25%)	438 (83.75%)
Shigella infection	66 (12.62%)	457 (87.38%)
*E. coli* infection	340 (65.01%)	183 (34.99%)
*Staphylococcus aureus* infection	131 (25.05%)	392 (74.95%)
Tuberculosis	389 (74.38%)	134 (25.62%)
Anthrax	340 (65.01%)	183 (34.99%)
Cysticercosis	19 (3.63%)	504 (96.37%)
Cyclospora	13 (2.49%)	510 (97.51%)
Teniasis	129 (24.67%)	394 (75.33%)
3. Transmission of infection?		
Raw/undercooked meat	465 (88.91%)	58 (11.09%)
Raw/unpasteurized milk and milk products	396 (75.72%)	127 (24.28%)
Raw egg	310 (59.27%)	213 (40.73%)
Contaminated water	415 (79.35%)	108 (20.65%)
Infected animals (farm)	311 (59.46%)	212 (40.54%)
Infected pet animals	213 (40.73%)	310 (59.27%)
Restaurant salad	151 (28.87%)	372 (71.13%)
4. Which statements about surveillance and reporting are accurate in controlling food‐borne zoonoses?		
Reporting unusual disease outbreaks in animals or humans helps with early detection and prevention	445 (85.09%)	78 (14.91%)
Integrated data from human health, animal health, and the environment (One Health surveillance) enable the effective monitoring of zoonotic threats	351 (67.11%)	172 (32.89%)
Informal community information (e.g., talking with neighbors) is more reliable than official veterinary reports	205 (39.20%)	318 (60.80%)
Surveillance only matters once human cases have already emerged	133 (25.43%)	390 (74.57%)
5. Which preventive strategies help control zoonotic diseases at the farm or community level?		
Vaccinating animals (when available)	458 (87.57%)	65 (12.43%)
Implementing farm biosecurity (e.g., limiting animal contact and proper waste disposal)	399 (76.29%)	124 (23.71%)
Ensuring veterinary treatment and not consuming meat from sick animals	316 (60.42%)	207 (39.58%)
Disposing of animal birth materials or aborted fetuses safely	214 (40.92%)	309 (59.08%)

### 3.3. Attitudes of Farmers Toward Food‐Borne Zoonoses

The attitudes of farmers toward FBDs are shown in Table 3. Approximately 89.3% (*n* = 467) of respondents supported the view that FBDs pose a serious public health threat, with approximately 83.4% (*n* = 436) believing that the use of antibiotics in animals should be regulated to safeguard human health. Approximately 88.5% of the participants believed that the government should enforce stronger food safety regulations. Approximately 85.3% of the participants agreed that close interaction with livestock increases the risk of contracting animal‐borne diseases. In addition, 90.6% (*n* = 474) of participants reported that training programs improve their knowledge of zoonoses, and 85.9% (*n* = 449) agreed that protective gear is important. Over half (89.1%, *n* = 466) agreed that safe food practices are essential for preventing FBDs. With respect to preventive measures, 84.5% (*n* = 442) believed that thoroughly cooking meat, milk, and eggs prevents food‐borne illness, whereas 91.3% (*n* = 477) disagreed that consuming raw milk, meat, and eggs is safe.

**TABLE 3:** Farmers’ attitudes toward food‐borne diseases (*n* = 523), presented as frequencies and percentages.

Variables	Strongly agree	Agree	Neutral	Disagree	Strongly disagree
Antibiotic use in animals should be regulated to protect humans	270 (51.63%)	166 (31.74%)	65 (12.43%)	10 (1.91%)	12 (2.29%)
Food‐borne zoonotic diseases are a serious public health threat	246 (47.04%)	221 (42.26%)	50 (9.56%)	5 (0.96%)	1 (0.19%)
The government should enforce stronger food safety regulations	268 (51.24%)	195 (37.28%)	56 (10.71%)	2 (0.38%)	2 (0.38%)
Working closely with livestock increases my risk of catching diseases from animals	205 (39.20%)	241 (46.08%)	63 (12.05%)	11 (2.10%)	3 (0.57%)
Training programs would improve people’s knowledge of zoonoses	253 (48.37%)	221 (42.26%)	43 (8.22%)	5 (0.96%)	1 (0.19%)
Protective gear is essential	210 (40.15%)	239 (45.70%)	63 (12.05%)	7 (1.34%)	4 (0.76%)
Safe food practices can prevent zoonotic diseases	268 (51.24%)	198 (37.86%)	45 (8.60%)	9 (1.72%)	3 (0.57%)
Cooking meat, milk, and eggs thoroughly (until there is no pink inside) prevents food‐borne illness	234 (44.74%)	208 (39.77%)	48 (9.18%)	7 (1.34%)	26 (4.97%)
Consuming raw milk, meat, and eggs is safe	12 (2.29%)	15 (2.87%)	19 (3.63%)	147 (28.11%)	330 (63.10%)

### 3.4. Practices of Participants Toward Food‐Borne Zoonoses

Table 4 summarizes the preventive and control practices employed by farmers to combat FBDs. Approximately 63.1% (*n* = 330) of participants reported never slaughtering sick animals or consuming meat in the field. The majority of the participants reported good hygiene practices, with 72.7% (*n* = 380) always washing their hands before food preparation and 75.7% (*n* = 396) always washing raw vegetables before consumption. A total of 42.3% (*n* = 221) of the participants sometimes used gloves or masks when handling raw meat or sick animals. More than half of the participants (*n* = 308, 58.9%) separated raw meat from ready‐to‐eat food during storage. Similarly, 68.3% (*n* = 357) of the participants never sold meat from sick animals to the public. However, only approximately one‐third of the participants (*n* = 169, 32.3%) consistently informed their family or community about safe food practices. In the case of street food, more than half (*n* = 311, 59.5%) of the respondents reported that they sometimes allow children to eat it, and nearly two‐thirds (*n* = 342, 65.4%) of them sought medical advice after being sick from outside food. Approximately three‐quarters of the participants reported boiling milk before consumption (78.4%, *n* = 410) and checking the expiration dates of meat or milk when purchasing from shops (75.1%, *n* = 393).

**TABLE 4:** Reported practices of farmers related to food‐borne disease (*n* = 523) prevention and control in Bangladesh (*n* = 523), presented as frequencies and percentages.

Variables	Always	Sometimes	Never
Slaughtering sick animals in the field and consuming the meat with others in the field	81 (15.49%)	112 (21.41%)	330 (63.10%)
Do you wash your hands before preparing food?	380 (72.66%)	140 (26.77%)	3 (0.57%)
Do you wash vegetables before eating raw?	396 (75.72%)	117 (22.37%)	10 (1.91%)
Have you ever taken your pet to a veterinary clinic for treatment?	197 (37.67%)	232 (44.36%)	94 (17.97%)
Do you use gloves/mask when handling raw meat or sick animals?	104 (19.89%)	221 (42.26%)	198 (37.86%)
Do you separate raw meat from ready‐to‐eat food during storage?	308 (58.89%)	192 (36.71%)	23 (4.40%)
Selling of sick animals’ meat to the public	45 (8.60%)	121 (23.14%)	357 (68.26%)
Do you inform others in your family/community about safe food practices?	169 (32.31%)	293 (56.02%)	61 (11.66%)
Do you consume raw/unpasteurized milk?	37 (7.07%)	96 (18.36%)	390 (74.57%)
Do you consume raw or undercooked meat?	25 (4.78%)	61 (11.66%)	437 (83.56%)
Purchasing veterinary drugs without a prescription	52 (9.94%)	52 (9.94%)	52 (9.94%)
Do you allow children to eat street food?	71 (13.58%)	311 (59.46%)	141 (26.96%)
Do you seek medical advice when sick after eating outside food?	151 (28.87%)	342 (65.39%)	30 (5.74%)
How often do you boil milk before drinking?	410 (78.39%)	107 (20.46%)	6 (1.15%)
Do you check the expiry date of meat/milk when buying from shops?	393 (75.14%)	116 (22.18%)	14 (2.68%)

### 3.5. Accessibility of Farmers Regarding Food‐Borne Zoonoses

**FIGURE 1:**
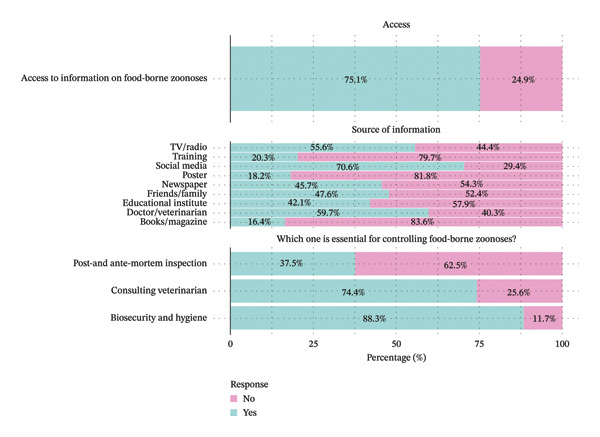
Sources of information and access to knowledge regarding food‐borne diseases among farmers in Bangladesh, including channels of communication and awareness of control measures.

Most farmers have access to information on FBDs, with approximately three‐fourths (75.1%) reporting such access (Figure 1). Regarding sources of information, farmers most commonly relied on social media (70.6%), followed by doctors or veterinarians (59.7%) and TV/radio (55.6%), whereas books or magazines were the least common source (16.4%). Moreover, 88.3% of the participants reported biosecurity and hygiene as the most essential measures for controlling FBDs, whereas more than one‐third of the farmers mentioned postmortem and antemortem inspection as crucial tools for controlling FBDs.

### 3.6. Levels of KAPs Regarding Food‐Borne Zoonoses

The study evaluated the levels of KAPs related to FBDs among 523 farmers. The findings revealed that only 8.8% (*n* = 46) demonstrated good knowledge of FBDs, whereas 35.8% (*n* = 187) had moderate knowledge and 55.5% (*n* = 290) had poor knowledge. In terms of attitudes, 59.3% (*n* = 310) displayed positive attitudes, whereas for practices, just over one‐third (*n* = 200, 38.2%) followed good practices, and the remaining 61.8% (*n* = 323) followed poor practices (Table 5).

**TABLE 5:** Overall levels of KAPs regarding food‐borne diseases among farmers in Bangladesh (*n* = 523).

Variables		*N* = 523^1^	95% CI
Knowledge level	Good	46 (8.80%)	6.6%, 12%
	Moderate	187 (35.76%)	32%, 40%
	Poor	290 (55.45%)	51%, 60%
Attitude level	Negative	2 (0.38%)	0.07%, 1.5%
	Positive	310 (59.27%)	55%, 63%
	Uncertain	211 (40.34%)	36%, 45%
Practice level	Good	200 (38.24%)	34%, 43%
	Poor	323 (61.76%)	57%, 66%

Abbreviation: CI = confidence interval.

^1^ *n* (%).

### 3.7. Effect of Sociodemographics on KAPs Regarding Food‐Borne Zoonoses

The factors associated with farmers’ levels of KAPs regarding FBDs are shown in Table 6. The study revealed that older participants (over 45 years old) and males generally demonstrated moderate knowledge and a positive attitude, whereas younger participants (18–30 years old) and females exhibited better practices. Similarly, farmers with higher levels of education demonstrated better knowledge and a more positive attitude; however, their practices were poorer than those of farmers with primary or secondary education. Family size was significantly associated with knowledge and attitudes, whereas marital status and training on food safety and hygiene were significantly associated with KAPs. In contrast, religion was not significantly associated with KAPs.

**TABLE 6:** Association between sociodemographic factors and KAP levels regarding food‐borne diseases among farmers, analyzed using chi‐square tests.

Variables		Knowledge				Attitude				Practices		
		Good	Moderate	Poor	*p* value	Negative	Positive	Uncertain	*p* value	Good	Poor	*p* value
Age	> 45	2 (2.15%)	47 (50.54%)	44 (47.31%)	**<** **0.001**	0 (0.00%)	58 (62.37%)	35 (37.63%)	0.7	30 (32.26%)	63 (67.74%)	**0.048**
	18–30	39 (13.64%)	97 (33.92%)	150 (52.45%)		2 (0.70%)	172 (60.14%)	112 (39.16%)		123 (43.01%)	163 (56.99%)	
	31–45	5 (3.47%)	43 (29.86%)	96 (66.67%)		0 (0.00%)	80 (55.56%)	64 (44.44%)		47 (32.64%)	97 (67.36%)	
Gender	Female	5 (3.73%)	29 (21.64%)	100 (74.63%)	**<** **0.001**	1 (0.75%)	62 (46.27%)	71 (52.99%)	**<** **0.001**	53 (39.55%)	81 (60.45%)	0.7
	Male	41 (10.54%)	158 (40.62%)	190 (48.84%)		1 (0.26%)	248 (63.75%)	140 (35.99%)		147 (37.79%)	242 (62.21%)	
Education	Higher	44 (16.60%)	122 (46.04%)	99 (37.36%)	**<** **0.001**	2 (0.75%)	189 (71.32%)	74 (27.92%)	**<** **0.001**	102 (38.49%)	163 (61.51%)	0.14
	Illiterate	0 (0.00%)	1 (3.57%)	27 (96.43%)		0 (0.00%)	10 (35.71%)	18 (64.29%)		5 (17.86%)	23 (82.14%)	
	Primary	1 (1.33%)	18 (24.00%)	56 (74.67%)		0 (0.00%)	32 (42.67%)	43 (57.33%)		31 (41.33%)	44 (58.67%)	
	Secondary	1 (0.65%)	46 (29.68%)	108 (69.68%)		0 (0.00%)	79 (50.97%)	76 (49.03%)		62 (40.00%)	93 (60.00%)	
Family members	> 6	4 (8.00%)	25 (50.00%)	21 (42.00%)	**0.025**	1 (2.00%)	27 (54.00%)	22 (44.00%)	**0.004**	18 (36.00%)	32 (64.00%)	> 0.9
	1–3	5 (8.47%)	12 (20.34%)	42 (71.19%)		0 (0.00%)	24 (40.68%)	35 (59.32%)		23 (38.98%)	36 (61.02%)	
	4–6	37 (8.94%)	150 (36.23%)	227 (54.83%)		1 (0.24%)	259 (62.56%)	154 (37.20%)		159 (38.41%)	255 (61.59%)	
Marital status	Married	10 (3.57%)	100 (35.71%)	170 (60.71%)	**<** **0.001**	1 (0.36%)	152 (54.29%)	127 (45.36%)	**0.004**	100 (35.71%)	180 (64.29%)	**0.04**
	Others	1 (16.67%)	1 (16.67%)	4 (66.67%)		0 (0.00%)	1 (16.67%)	5 (83.33%)		0 (0.00%)	6 (100.00%)	
	Single	35 (14.77%)	86 (36.29%)	116 (48.95%)		1 (0.42%)	157 (66.24%)	79 (33.33%)		100 (42.19%)	137 (57.81%)	
Training	No	33 (8.17%)	161 (39.85%)	210 (51.98%)	**0.002**	0 (0.00%)	257 (63.61%)	147 (36.39%)	**<** **0.001**	132 (32.67%)	272 (67.33%)	**<** **0.001**
	Yes	13 (10.92%)	26 (21.85%)	80 (67.23%)		2 (1.68%)	53 (44.54%)	64 (53.78%)		68 (57.14%)	51 (42.86%)	
Religion	Hindu	6 (6.25%)	33 (34.38%)	57 (59.38%)	0.8	1 (1.04%)	57 (59.38%)	38 (39.58%)	0.2	32 (33.33%)	64 (66.67%)	0.4
	Muslim	40 (9.48%)	152 (36.02%)	230 (54.50%)		1 (0.24%)	248 (58.77%)	173 (41%)		167 (39.57%)	255 (60.43%)	
	Others	0 (0.00%)	2 (40.00%)	3 (60.00%)		0 (0.00%)	5 (100.00%)	0 (0.00%)		1 (20.00%)	4 (80.00%)	

*Note:* Significant values are in bold.

### 3.8. Factors Associated With Knowledge Level Regarding Food‐Borne Zoonoses

Univariate ordinal regression analysis revealed that several sociodemographic factors significantly influence farmers’ knowledge of FBDs (Table 7). Compared with females, male farmers were found to have substantially greater odds of possessing good knowledge (OR = 3.08; 95% CI: 2.01–4.80; *p* < 0.001). Education emerged as a strong determinant of knowledge, with illiterate (OR = 0.02; 95% CI: 0.00–0.10; *p* < 0.001), primary (OR = 0.18; 95% CI: 0.10–0.32; *p* < 0.001), and secondary (OR = 0.23; 95% CI: 0.15–0.35; *p* < 0.001) farmers showing significantly lower odds of good knowledge than those with higher education. Farmers aged 31–45 years presented significantly lower odds of having good knowledge than those over 45 years (OR = 0.52; 95% CI: 0.31–0.86; *p* = 0.011). Smaller family size (1–3 persons) was also associated with lower knowledge levels (OR = 0.35; 95% CI: 0.16–0.74; *p* = 0.007). Compared with married individuals, single farmers presented greater odds of having good knowledge (OR = 1.84; 95% CI: 1.31–2.59; *p* < 0.001). Interestingly, farmers who received training had poorer knowledge than those without training did (OR = 0.59; 95% CI: 0.38–0.90; *p* = 0.016).

**TABLE 7:** Factors associated with knowledge levels regarding food‐borne diseases among farmers in Bangladesh, analyzed using univariate logistic regression to assess crude associations, followed by multivariate regression.

Characteristics		OR (95% CI)	*p* value
Age	> 45 years	—	
	18–30 years	1.04 (0.67, 1.62)	0.9
	31–45 years	0.52 (0.31, 0.86)	**0.011**
Gender	Female	—	
	Male	3.08 (2.01, 4.80)	**<** **0.001**
Education	Higher	—	
	Illiterate	0.02 (0.00, 0.10)	**<** **0.001**
	Primary	0.18 (0.10, 0.32)	**<** **0.001**
	Secondary	0.23 (0.15, 0.35)	**<** **0.001**
Family	> 6 persons	—	
	1–3 persons	0.35 (0.16, 0.74)	**0.007**
	4–6 members	0.67 (0.39, 1.17)	0.2
Marital	Married	—	
	Others	0.97 (0.13, 5.18)	> 0.9
	Single	1.84 (1.31, 2.59)	**<** **0.001**
Training	No	—	
	Yes	0.59 (0.38, 0.90)	**0.016**
Religion	Hindu	—	
	Muslim	1.25 (0.81, 1.96)	0.3
	Others	0.89 (0.12, 4.87)	0.9

*Note:* Significant values are in bold.

Abbreviations: CI = confidence interval; OR = odds ratio.

### 3.9. Factors Associated With Attitudes Toward Food‐Borne Zoonoses

Univariate ordinal regression analysis revealed several sociodemographic factors significantly associated with farmers’ attitudes toward FBDs (Table 8). Compared with females, male farmers were significantly more likely to have a positive attitude (OR = 2.05; 95% CI: 1.38–3.05; *p* < 0.001). Education level exhibited a strong positive association, where participants with no formal education (OR = 0.23; 95% CI: 0.10–0.51; *p* < 0.001), primary education (OR = 0.31; 95% CI: 0.18–0.52; *p* < 0.001), and secondary education (OR = 0.43; 95% CI: 0.28–0.64; *p* < 0.001) had significantly lower odds of having a positive attitude than those with higher education. Although age group, family size, and marital status were not significantly associated (*p* > 0.05), single respondents were more likely to have a positive attitude than married individuals were (OR = 1.65; 95% CI: 1.15–2.36; *p* = 0.006). Additionally, farmers who had received training presented significantly lower odds of having a poor attitude than did those without training (OR = 0.45; 95% CI: 0.29–0.68; *p* < 0.001), highlighting the beneficial influence of training programs on farmers’ awareness and attitudes toward FBDs.

**TABLE 8:** Factors associated with attitudes toward food‐borne diseases among farmers in Bangladesh, assessed using univariate logistic regression for crude associations and multivariate regression.

Characteristics		OR (95% CI)	*p* value
Age	> 45 years	—	
	18–30 years	0.90 (0.55, 1.45)	0.7
	31–45 years	0.76 (0.44, 1.28)	0.3
Gender	Female	—	
	Male	2.05 (1.38, 3.05)	**<** **0.001**
Education	Higher	—	
	Illiterate	0.23 (0.10, 0.51)	**<** **0.001**
	Primary	0.31 (0.18, 0.52)	**<** **0.001**
	Secondary	0.43 (0.28, 0.64)	**<** **0.001**
Family	> 6 persons	—	
	1–3 persons	0.61 (0.28, 1.30)	0.2
	4–6 members	1.47 (0.81, 2.65)	0.2
Marital	Married	—	
	Others	0.19 (0.02, 1.11)	0.10
	Single	1.65 (1.15, 2.36)	**0.006**
Training	No	—	
	Yes	0.45 (0.29, 0.68)	**<** **0.001**

*Note:* Significant values are in bold.

Abbreviations: CI = confidence interval; OR = odds ratio.

### 3.10. Factors Associated With the Practice Level of Food‐Borne Zoonoses

The factors influencing farmers’ practices toward FBDs are summarized in Table 9. Although younger farmers aged 18–30 years tended to adopt better practices than those aged over 45 years (OR = 1.58; 95% CI: 0.97–2.62; *p* = 0.068), the association was not statistically significant. Education level showed a mixed pattern, with only illiterate farmers having considerably lower odds of good practices than those with higher education (OR = 0.35; 95% CI: 0.11–0.87; *p* = 0.038). Moreover, farmers who received training were nearly three times more likely to demonstrate good practices than those without training (OR = 2.75; 95% CI: 1.81–4.19; *p* < 0.001). Gender, family size, and religion were not significantly associated with farmers’ practice levels (*p* > 0.05).

**TABLE 9:** Factors associated with preventive practices regarding food‐borne diseases among farmers in Bangladesh, evaluated using univariate logistic regression followed by multivariate regression analyses.

Characteristics		OR (95% CI)	*p* value
Age	> 45 years	—	
	18–30 years	1.58 (0.97, 2.62)	0.068
	31–45 years	1.02 (0.58, 1.79)	> 0.9
Gender	Female	—	
	Male	0.93 (0.62, 1.39)	0.7
Education	Higher	—	
	Illiterate	0.35 (0.11, 0.87)	**0.038**
	Primary	1.13 (0.66, 1.89)	0.7
	Secondary	1.07 (0.71, 1.60)	0.8
Family	> 6 persons	—	
	1–3 persons	1.14 (0.52, 2.49)	0.7
	4–6 members	1.11 (0.61, 2.08)	0.7
Training	No	—	
	Yes	2.75 (1.81, 4.19)	**< 0.001**
Religion	Hindu	—	
	Muslim	1.31 (0.83, 2.11)	0.3
	Others	0.50 (0.03, 3.55)	0.5

*Note:* Significant values are in bold.

Abbreviations: CI = confidence interval; OR = odds ratio.

## 4. Discussion

Food‐borne zoonoses remain a significant public health concern in low‐ and middle‐income countries, where animal‐derived foods are handled in informal settings. This study presents the first comprehensive investigation of Bangladeshi farmers’ KAPs regarding FBDs from a One Health (OH) perspective. These findings indicate that although most farmers have heard of zoonotic infections, their detailed understanding of specific pathogens, transmission routes, and preventive measures is limited. The pattern revealed here, with a positive attitude but poor practice, reflects the characteristic gap between awareness and behavioral implementation previously noted in South Asian KAP studies [27, 36–38].

The demographic profile revealed that the majority of the participants were male and aged 18–45 years, which is comparable to the national farming demography of Bangladesh [39, 40]. Such gender dominance has also been reported among livestock farmers in Nigeria and Ethiopia [41, 42], where men traditionally engage in market‐oriented farming and slaughter activities, exposing them more to zoonotic risks. However, several studies in Sub‐Saharan Africa and parts of South Asia have reported a predominance of female livestock farmers [43, 44], particularly in smallholder or backyard systems where women are primarily responsible for animal care and milking. This contrast highlights the influence of local gender roles and labor division on zoonotic exposure and risk perception. Education varied widely, with a considerable proportion having secondary education, whereas only a small percentage had higher education. This heterogeneity likely influences comprehension of disease transmission and risk behavior, as evidenced by the significant association between education level and knowledge score. Farmers lacking formal education demonstrated the lowest levels of awareness and need for training; this pattern is consistent with previous findings from India and Ethiopia [45].

Although 90% of the respondents had heard of FBDs, this figure is higher than that reported in a study conducted by Otuh et al. [46], who reported 72% of the respondents. Only a small proportion could correctly identify the causative agents beyond common bacterial infections. *Salmonella* spp. and *E. coli* are the most frequently recognized pathogens, whereas protozoal and viral pathogens are rarely mentioned. This indicates a selective familiarity with well‐publicized diseases. The under‐recognition of parasitic and viral zoonoses mirrors observations among farmers in Nepal and Bangladesh [27, 47]. Misconceptions about food contamination channels persist; most farmers associate infection solely with raw meat or milk but underestimate indirect exposures such as contaminated vegetables or restaurant foods. These results highlight the narrow perception of “animal‐origin” diseases, often excluding environmental and cross‐contamination pathways integral to the OH framework. A study by Abebaw and Assefie [28] reported that most farmers recognized that food from infected animals and unpasteurized milk were the primary sources of food‐borne infections. Most farmers recognized that preventing FBDs primarily involves vaccination, enforcing farm biosecurity measures, providing veterinary care, and avoiding the consumption of meat from sick animals. Similar findings were also reported by Abebaw and Assefie [28]. These findings indicate that farmers are generally aware of the critical measures required to mitigate FBDs.

Despite these gaps, the respondents demonstrated encouraging attitudes toward FBDs. More than three‐fourths of the farmers in our study acknowledged that regulating antibiotic use in animals is essential for safeguarding human health. The results demonstrate strong awareness among farmers regarding antibiotic use and public health implications. Our study indicates that more than 85% of farmers agreed that FBDs are serious public health threats, which is higher than that reported in existing studies in Nepal (72%) by Subedi et al. [48] and in Ethiopia (45.1%) by Abunna et al. [49]. Given the limited awareness, particularly among illiterate and rural farmers, there is a pressing need for targeted training and outreach programs [48]. The effectiveness of such interventions may be enhanced by leveraging farmers’ underlying motivations, as previous studies among Bangladeshi butchers and dairy farmers have shown that positive attitudes are often influenced by economic considerations and concerns for family health [50]. This suggests that aligning educational programs with these motivating factors may improve their impact and adoption. A large majority (over 80%) of the farmers supported the idea that robust enforcement of food safety regulations is crucial for ensuring food quality and preventing zoonotic disease transmission. This level of awareness indicates that farmers are not only conscious of food‐borne risks but also recognize the role of effective governance in ensuring safe food production. The strong support observed in our study suggests a favorable environment for policy implementation and farmer participation in regulatory compliance programs. A large majority of farmers (over 90%) believed that zoonotic disease training programs could strengthen their understanding of disease prevention, thereby protecting both their families and livestock. This positive attitude provides strong evidence that education plays a key role in disease prevention strategies. A large proportion of farmers recognized that thoroughly cooking meat and eggs, adhering to safe food‐handling practices, and refraining from consuming raw animal products such as milk, meat, and eggs are essential measures to prevent FBDs. These practices are crucial for breaking the transmission cycle of many FBDs, including *Salmonella* spp., *Campylobacter* spp., and *Brucella* spp. [51, 52].

Although the farmers in this study demonstrated good knowledge and positive attitudes toward FBDs, their actual practices revealed areas that needed improvement. Encouragingly, one‐third of the farmers always washed their hands before preparing food, washed vegetables before eating them raw, boiled milk before drinking, and checked the expiration date of meat before purchasing it. However, these findings are based on self‐reported data and may be subject to recall and social desirability bias, potentially leading to overestimation of these practices. These preventive measures are essential for reducing the risk of FBDs and indicate that many farmers are applying their knowledge in daily life. These findings are consistent with previous studies that reported relatively high levels of safe food handling practices among farmers [43, 49, 53, 54].

However, several risky practices persist despite this good level of knowledge. Notably, 15.5% of respondents reported slaughtering sick animals in the field and consuming the meat, while over a quarter admitted to selling such meat to the public. Although a majority (63.1%) reported never engaging in these practices, the proportion of farmers who still do so remains a significant concern. These behaviors pose substantial public health risks, as consuming or distributing meat from diseased animals can facilitate the transmission of pathogens such as *Brucella* spp., *Bacillus anthracis*, and *Salmonella* spp.

Only a limited number of people consistently use personal protective equipment, such as gloves or masks, when handling raw meat or sick animals, highlighting a clear gap between knowledge and actual practice. More than half of the respondents reported sharing food safety information with their families or communities only occasionally, indicating weak community‐level health communication.

Furthermore, several practices may contribute to the broader transmission of FBDs. Some pathogens can spread further through close human‐to‐human contact, thereby increasing the potential for wider community transmission [52]. Similarly, only 37.7% reported regularly seeking veterinary care for their animals, indicating limited utilization of formal veterinary services. Additionally, 9.94% reported purchasing veterinary drugs without a prescription, raising concerns about misuse and potential contributions to antimicrobial resistance. Similar patterns have been documented in previous studies, where low engagement with veterinary services and inappropriate drug use were identified as key gaps in disease control strategies [28].

Access to information and its sources plays a crucial role in shaping farmers’ KAPs concerning FBDs [55]. In this study, more than three‐quarters of the farmers reported having access to information regarding FBDs. Among sources of information, social media is the most commonly used, followed by television, radio, advice from doctors or veterinarians, and newspapers. This contrasts with the study by Abunna et al. [49] in Ethiopia, where community sources were reported as the most frequently used, followed by radio and newspapers, whereas social media accounted for only a negligible portion. The difference may be due to variations in regional access to digital platforms. These results highlight the growing importance of digital channels for disseminating health information and suggest that leveraging social media could be an effective strategy to improve farmers’ knowledge and practices in food safety and zoonotic disease prevention.

With respect to KAP levels, the findings indicate substantial gaps in farmers’ KAPs regarding FBDs. Only a small proportion of farmers demonstrated good knowledge of FBDs, and a moderate share exhibited acceptable knowledge; the majority had poor knowledge. In contrast, more than half of the participants reported positive attitudes, yet only a minority consistently followed preventive practices, highlighting a gap between awareness and attitudes and actual practices. Farmers with higher levels of education demonstrated greater knowledge and a more positive attitude. These trends were also reported by Ahmed et al. [27]. Older participants and males generally demonstrated moderate knowledge and maintained a positive attitude toward FBDs, suggesting that experience may contribute to awareness but does not necessarily translate into best practices. In contrast, younger participants and females tended to exhibit better preventive practices, indicating that this group may be more receptive to adopting safe behaviors despite having comparatively lower or moderate knowledge and attitudes.

### 4.1. Limitations

As a cross‐sectional study, our research has several limitations. Given that the study is questionnaire‐based, it may be subject to bias during data collection. Additionally, the use of multiple‐choice questions makes some degree of response bias unavoidable; however, this bias may have been partially mitigated by the stringent criteria used to construct the knowledge scores. As the study included farmers who had prior experience of reporting animal or poultry diseases to veterinary hospitals, the sample may not be fully representative of the broader farming community. This inclusion criterion may have introduced selection bias, as such farmers are more likely to have greater awareness and engagement with animal health services.

### 4.2. Implications

The findings of this study emphasize the urgent need for comprehensive policy interventions to increase farmers’ KAPs concerning FBDs in Bangladesh. Policymakers should strengthen farmer education through targeted training, awareness campaigns, and veterinary extension programs focused on zoonotic disease prevention, hygienic food handling, and biosecurity practices. Integrating an OH approach into national livestock and public health frameworks is vital to promote collaboration among the agriculture, health, and environmental sectors. The lack of interdisciplinary training for veterinarians and human physicians currently hampers effective collaboration in zoonotic disease control. Therefore, policymakers must prioritize health system reforms and implement integrated policies that link human, animal, and environmental health. Establishing interdisciplinary and multidisciplinary training programs, fostering collaborative research, and building effective communication networks across sectors will enhance coordinated responses. Furthermore, ensuring fair resource allocation, investing in disease prevention at the source, and encouraging leadership that values cross‐sectoral contributions are essential for sustainable zoonotic disease prevention and improved public health outcomes in Bangladesh.

## 5. Conclusion

The results of this study indicate that most farmers in the study area have limited awareness of FBD transmission, prevention, and control. Several problems arose from farmers’ attitudes and practices toward zoonoses. For example, a lack of awareness about washing hands before and after handling animals, handling food without gloves, not using protective clothing, and consuming or selling products from infected animals can all contribute to the transmission of food‐borne and zoonotic pathogens. These unsafe practices highlight critical knowledge and behavioral gaps that need to be addressed. Targeted educational interventions, comprehensive training programs, and consistent supervision through field extension services are essential strategies to increase farmer knowledge and practices and reduce the risk of zoonotic disease transmission.

**Nomenclature**
CI: Confidence interval
OR: Odds ratio
OH: One Health
WHO: World Health Organization
FBD: Food‐borne disease
KAPs: Knowledge, attitudes, and practices

## Author Contributions

Md Jisan Ahmed: writing–review and editing, writing–original draft, visualization, validation, supervision, software, resources, project administration, methodology, investigation, formal analysis, data curation, and conceptualization; Md Ismile Hossain Bhuiyan, Prajwal Bhandari, and Ritu Chalise: writing–review and editing and writing–original draft; Sazzad Bin Yosuf, Md Easin, Soumik Sarker, Md Rayhan Ullah Rahi, Hridoy Chakrabarty, Prottoy Basak, Ajoy Paul, and Rahat Nahiyan Ramim: data curation and validation; Delower Hossain: writing–review and editing, writing–original draft, data curation, validation, supervision, and resources.

## Funding

This study received no external or internal funding.

## Consent

The authors have nothing to report.

## Conflicts of Interest

The authors declare no conflicts of interest.

## Supporting Information

Additional supporting information can be found online in the Supporting Information section.

## Supporting information


**Supporting Information** Supporting File 1: questionnaire.

## Data Availability

The datasets used and/or analyzed during the current study are available from the corresponding author upon reasonable request.
