# Supplementary material for: Food‐Borne Diseases in Bangladesh: First Assessment of Knowledge, Attitudes, and Practices of Farmers
Source: Vet Med Int. 2026 Jun 30;2026:3617712. doi: 10.1155/vmi/3617712 (PMC13318510; doi:10.1155/vmi/3617712)
Supplement: Supplementary file 1 — Supporting Information Supporting File 1: questionnaire. [file VMI-2026-3617712-s001.pdf]

Food-borne Zoonosis among farmers in Bangladesh

Record your current location

latitude (x.y °)

longitude (x.y °)

altitude (m)

accuracy (m)

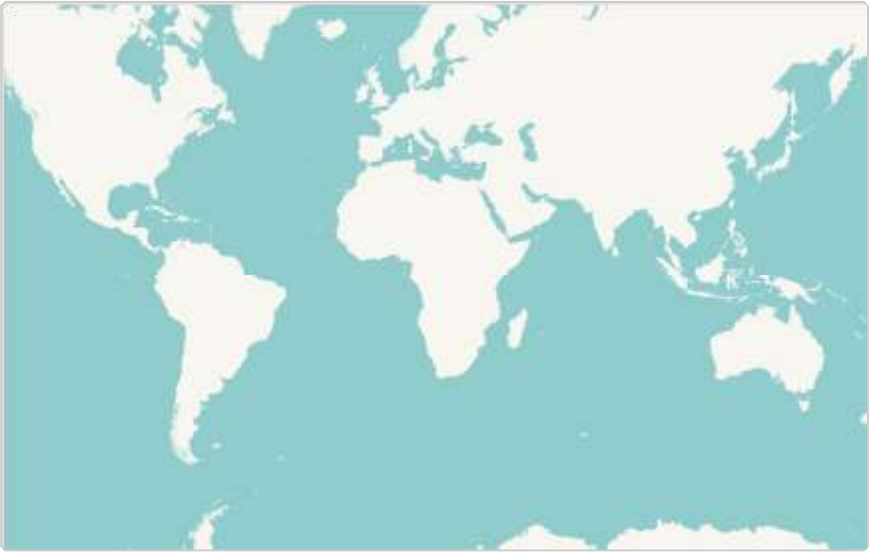

Data Collector (Sentence Case)

Part 1. Sociodemographic Information

1. Age (Years)

2. Gender

- ◯ Male
- ◯ Female

3. Education

- ◯ Illiterate
- ◯ Primary
- ◯ Secondary
- ◯ Higher

4. Monthly Income

5. Family Members

6. Marital Status

- ◯ Single
- ◯ Married
- ◯ Others

8. Training on Food safety and Hygiene

- ◯ Yes
- ◯ No

7. Religion

- ◯ Hindu
- ◯ Muslim
- ◯ Others

Part 2: Knowledge Information

1. Have you heard of 'Food-borne zoonotic diseases'?

- ◯ Yes
- ◯ No

2. Name of food-borne zoonoses (All are correct options)

- ☐ Novo Virus
- ☐ Salmonella
- ☐ Campylobacter
- ☐ Clostridium
- ☐ Listeria
- ☐ Shigella
- ☐ E. coli
- ☐ Staphylococcus
- ☐ TB
- ☐ Anthrax
- ☐ Cysticercosis
- ☐ Cyclospora
- ☐ Taeniasis

3. Transmission of Infection (All are correct options)

- [ ] Raw/undercooked meat
- [ ] Raw/unpasteurized milk and milk products
- [ ] Raw egg
- [ ] Contaminated water
- [ ] Infected animals (farm)
- [ ] Infected Pet animals
- [ ] Restaurant Salad

4. Which statements about surveillance and reporting are accurate in controlling foodborne zoonoses?

- [ ] Reporting unusual disease outbreaks in animals or humans helps with early detection and prevention. (Correct)
- [ ] Integrated data from human health, animal health, and the environment (One Health surveillance) helps monitor zoonotic threats effectively. (Correct)
- [ ] Informal community information (e.g., talking with neighbors) is more reliable than official veterinary reports. (Incorrect)
- [ ] Surveillance only matters once human cases have already emerged. (Incorrect)

5. Which preventive strategies help control zoonotic diseases at the farm or community level? (All are correct options)

- [ ] Vaccinating animals (when available)
- [ ] Implementing farm biosecurity (e.g., limiting animal contact, proper waste disposal)
- [ ] Ensuring veterinary treatment and not consuming meat from sick animals
- [ ] Disposing of animal birth materials or aborted fetuses safely

Part 3: Attitude Information

Part 3: Attitude Information	Strongly Agree	Agree	Neutral	Disagree	Strongly disagree
Antibiotic use in animals should be regulated to protect humans	○	○	○	○	○
Foodborne zoonotic diseases are a serious public health threat	○	○	○	○	○
The government should enforce stronger food safety regulations	○	○	○	○	○
Working closely with livestock increases my risk of catching diseases from animals.	○	○	○	○	○
Training programs would improve people's knowledge of zoonoses	○	○	○	○	○
Protective gear is essential.	○	○	○	○	○
Safe food practices can prevent zoonotic diseases	○	○	○	○	○
Cooking meat, milk, and egg thoroughly (until there is no pink inside) prevents foodborne illness.	○	○	○	○	○

**Drinking raw milk, meat, and eggs is safe.**	○	○	○	○	○

Part 4: Practice Information

**Part 4: Practice Information**	Always	Sometimes	Never
**Slaughtering sick animals in the field and consuming the meat with others in the field**	○	○	○
**Do you wash your hands before preparing food?**	○	○	○
**Do you wash vegetables before eating raw?**	○	○	○
**Have you ever had your animal treated in veterinary clinics?**	○	○	○
**Do you use gloves/mask when handling raw meat or sick animals?**	○	○	○
**Do you separate raw meat from ready-to-eat food during storage?**	○	○	○
**Selling of sick animals' meat to the public**	○	○	○
**Do you inform others in your family/community about safe food practices?**	○	○	○
**Do you consume raw/unpasteurized milk?**	○	○	○
**Do you consume raw or undercooked meat?**	○	○	○
**Purchasing veterinary drugs without a prescription**	○	○	○
**Do you allow children to eat street food?**	○	○	○
**Do you seek medical advice when sick after eating outside food?**	○	○	○
**How often do you boil milk before drinking?**	○	○	○
**Do you check the expiry date of meat/milk when buying from shops?**	○	○	○

Part 5: Accessibility about Foodborne zoonoses

1. Access to information on foodborne zoonoses

- ○ Yes
- ○ No

2. Source of Information

- [ ] Training
- [ ] Social Media
- [ ] TV/Radio
- [ ] Newspaper
- [ ] Books/Magazine
- [ ] Poster
- [ ] Friends/Family
- [ ] Educational Institute
- [ ] Doctor/Veterinarian

3. Which one is essential for controlling food-borne zoonoses?

- [ ] Consulting Veterinarian
- [ ] Post- and ante-mortem inspection
- [ ] Biosecurity and hygiene
